# Evaluation of a New Inverse, Globally Convex Treatment Planning System Algorithm for Gamma Knife Radiation Surgery Within a Prospective Trial: Advantages and Disadvantages in Practical Application

**DOI:** 10.1016/j.adro.2022.101006

**Published:** 2022-06-29

**Authors:** Feline Heinzelmann, Moritz Budde, Irenäus A. Adamietz, Kevin Kröninger, Jan P. Boström

**Affiliations:** a Department of Physics, TU Dortmund University, Dortmund, Germany; b University Hospital at Ruhr-Universität Bochum, Gamma Knife Zentrum, Bochum, Germany; c Marien Hospital Herne, University Hospital at Ruhr-Universität Bochum, Clinic for Radiotherapy and Radiation Oncology, Herne, Germany

## Abstract

**Purpose:**

A new inverse planning software called IntuitivePlan (IP) based on a global convex optimization algorithm was adopted for the Gamma Knife radiation surgery. We investigated IP's suitability for daily clinical use and its applicability for different cerebral entities.

**Methods and Materials:**

For 230 target volumes, IP was tested in a prospective trial. The computed treatment plans were compared with conventional expert preplans, which included forward planning by the expert and local internal optimization. Based on the same dose constraints, we used the default settings for the inverse calculation of the treatment plans. Plan quality metrics such as the Paddick conformity index were compared for both planning techniques with additional subdivisions into the 3 selectable IP planning strategies and different entity groups.

**Results:**

IP calculated treatment plans of quality similar to that of preplans created by expert planners. Some plan quality metrics, especially those related to conformity and dose gradient, attained statistically significantly higher scores combined with high coverage for the inversely generated plans except for the selectivity optimizing strategy. Normal brain volume receiving 10 Gy or 12 Gy or higher (*V*_1__0__Gy_ or *V*_1__2__Gy__)_ did not show significant differences for the coverage optimizing strategies. The IP software demonstrated significantly shorter planning times versus manual planning as well as greater numbers of isocenters, often associated with longer treatment times. In terms of total time, these differences almost balanced out again.

**Conclusions:**

Our results suggest that IP is advantageous for complex tumors. We observed general clinical significance for conformity and superiority for the selectivity optimizing strategy. In addition, the high-quality calculation from IP enables novices in the profession to achieve pre-treatment plans of a quality similar to that of expert planners. IP allows for optimizing the sparing of surrounding tissue and conformity for benign tumors within a short time. Thus, IP forms a solid basis for further planning on the treatment day.

## Introduction

In the early 1950s, Swedish neurosurgeon Lars Leksell introduced stereotactic radiation surgery to treat localized narrow lesions in the brain.^1^ Leksell used cross-firing photon beams instead of an open surgery procedure. Recently, the use of Leksell's approach, known as Gamma Knife (GK), has considerably increased and has been applied to treat benign and malignant brain tumors, vascular malformations, and functional disorders because of its high dose gradient and high precision.^2^, ^3^, ^4^

In contrast to other radiation therapies, in Gamma Knife radiation surgery, the standard technique for radiation treatment planning is forward planning, which includes the manual placement of isocenters. In the next step, the isocenters can be edited through the internal optimization in Leksell GammaPlan (LGP) software, optimizing the position, weight, and collimator configuration of all the shots in the target according to an objective function. However, this inverse dose planning often finds only the local and not the global minimum of the cost function.^5^ In addition, the time for treatment planning depends on the planner's experience. Therefore, an alternative or complement is being sought in the form of automated inverse dose planning, particularly for complex and irregularly shaped target volumes (TVs). The software IntuitivePlan (IP) with an inverse planning algorithm developed by a university start-up company presents such an alternative. It was conformité européenne marked for its use with Leksell Gamma Knife in June 2019. We were provided with this algorithm free of charge for study purposes. The software promises to find the global minimum by prescribing a dose to the target volume and specifying dose constraints for the organs at risk (OARs).^5^

The specific objective of this study was to examine the default performance of IP in comparison with the common expert planning technique in clinical routine. We conducted a prospective study with 117 cases of different cerebral diseases using both methods in each case. Furthermore, our research aimed to verify the applicability of IP for various diseases that occurred within the scope of this study.

## Methods and Materials

### Study assembly

In total, more than 100 planning examples with different cerebral diseases treated with Gamma Knife Perfexion were included in the prospective clinical trial. In the final analysis, the data of a total of 117 treatment cases were evaluated. The treated entities were classified as benign, malignant, functional, and vascular (Table 1). Cerebral singular and multiple metastases were counted among malignant disorders. Acoustic neuromas (ANs) and other neurinomas, pituitary adenomas, and meningiomas were classed as benign entities, whereas arteriovenous malformations (AVMs), fistulas, and cavernomas were categorized as vascular diseases and trigeminal neuralgias as functional disorders. The contouring of TVs and OARs was performed on T1- and T2-weighted 1-mm thin-layered 3-dimensional magnetic resonance imaging (MRI) images by Brainlab Elements (Anatomic Mapping 1.1, SmartBrush 3.0).

**Table 1:** Patient demographics

	n	$V_{50\;\%}$ in$\;cm^{3}$	$PD_{50\;\%}\;$in Gy
TVs	230	0.5500	18.0
Malign tumors	164	0.3625	20.0
Benign tumors	61	1.4780	14.0
Vascular diseases	4	1.3655	16.0
Functional diseases	1	0.0110	90.0

*Abbreviations:* PD_50%_ = median prescription dose for forward planning; TV = target volume; *V*_50%_ = median target volume.

### Forward planning with LGP

The forward planning was performed by expert planners with long-term experience as medical physics experts and 2 years’ experience in Gamma Knife therapy. The treatment planning system used in this study is called Leksell GammaPlan (version 11.3.1). With LGP's internal inverse planning feature, the plans were generally optimized. For small-sized metastases, only manual forward planning was used, mostly with one shot.

In general, the criteria for a good or acceptable plan are as follows: coverage of 0.95 to 1.00, selectivity of 0.7 to 1.00, a gradient index below 3.0, and beam-on time (BOT) between 10 and 120 minutes. These may deviate in the individual case, depending on medical reasons such as the disease and the patient's medical history. The coverage, for instance, is of primary importance for single metastases, whereas the selectivity and the irradiation time are decisive factors for ANs and multiple metastases, respectively.

### Inverse planning with IP

The cases were replanned through the inverse planning software IntuitivePlan (version 1.0) after the export of the patient data from LGP. According to the departmental clinical protocol, the same dose constraints as for the LGP planning were set up. The optimization strategies (“maximize coverage” with the options “favor selectivity” or “favor BOT” and “maximize selectivity”) were specifically applied for the case or entity. To compare the elementary performance of the new algorithm with the present treatment (forward) planning method, the default parameters for each strategy were not modified, and no other sophisticated functions were used in this study, such as 3-dimensional manipulation of the isodose surfaces. For patient treatment, the resulting shot configuration of IP plans generally need to be exported back to LGP. It is necessary to adjust the optimized prescription dose from IP to integers, which consequently affects the dose distribution and plan quality metrics, but to a slight extent. Metastases were predominantly optimized according to the two strategies focusing on coverage, whereas the strategies “maximize selectivity” and “maximize coverage, favor selectivity” were mainly applied to benign entities, vascular and functional diseases.

### Treatment plan evaluation

Both LGP and IP plans were preliminary and were based on nonstereotactic 3-dimensional MRI images with a simulated stereotactic frame. On the treatment day, the preplans were finally adjusted to the entity by coregistration with stereotactic tomography.

To compare the planning results from both treatment planning methods, or rather, to assess the benefit for the target and healthy tissue, the following parameters were evaluated: coverage, selectivity, gradient index (GI), Paddick conformity index (PCI),^6^ efficiency index (EI), BOT, planning or computational time ($t_{\text{plan}}$), total set-up time ($t_{\text{total}}$), number of shots (η_shots_), number of blocked sectors (η_blocked sectors_), prescription isodose (PI), minimum dose (*D*_min_), mean dose (*D*_mean_), maximum dose (*D*_max_), and for risk assessment for brain tissue and OARs, the volume of brain irradiated with 12 Gy (V_12 Gy_) or 10 Gy (V_10 Gy_), the mean brain dose (*D*_skull mean_), and the difference between the maximum dose and dose constraint in the respective OAR (*D*_OAR transgression_). The efficiency index proposed by Paddick^7^ considers the ratio of integral dose inside and outside the target:

$$\eta_{50\;\%}=\frac{useful\;energy}{total\;energy}=\frac{Integral\;Dose_{\text{TV}}}{Integral\;Dose_{\text{PIV}50\;\%}}=\frac{\int_{D\text{min}}^{D\text{max}}\text{TV}\;d\text{Dose}}{\int_{D\text{min}}^{D\text{max}}\text{PI}V_{50\;\%}d\text{Dose}\;},$$

where PIV_50%_ is the absolute volume of 50%of the PI. The efficiency index for multiple targets (Gη_12 Gy_) fixes the problem of the gradient index for nearby targets that can overlap in PIV_50%_^7^:

$$G\eta_{12\;\text{Gy}}=\frac{\sum_{n=\;2}^{N}Integral\;Dose_{TV_{n}\;}}{Global\;Integral\;Dose_{12\;\text{Gy}\;}}.$$

The efficiency indices η_50%_ and Gη_12 Gy_ need to be extracted from the dose-volume histograms for LGP. The EI for single targets is automatically calculated by IntuitivePlan.

One of the most relevant late toxic effects occurring after stereotactic radiation surgery is radionecrosis, which correlates with the *V*_12 Gy_ or *V*_10 Gy_.^8^ Likewise, we introduced the parameter *D*_OAR transgression_ in the respective OAR (brain stem, cochlea, optic chiasma, pituitary, trigeminal nerve, vestibular apparatus, or optical nerve). The sum of the BOT and planning time was described as *t*_total_. For the statistical *t-*tests, the times per case instead of per TV were considered for both the plan calculation time and the total time, because IP only reported the total calculation time for the cases with multiple target volumes. Because the skull contouring was not feasible with the available Brainlab Elements version, the mean brain dose was represented by the parameter *D*_skull mean_.

### Statistical analysis

Initially, we compared the IP strategies separately with LGP. Moreover, we investigated IP in terms of its applicability to malignant and benign diseases. To determine the statistical significance between the parameters of both planning methods, we conducted 2-sided paired samples *t-*tests^9^^,^^10^ with a significance level of 5% (α = .05). The null hypothesis stated that the mean values of LGP and IP did not differ (*μ*_LGP_ = *μ*_IP_).

With multiple testing, that is, running various statistical tests on the same sample, the overall risk that at least 1 of the tests becomes falsely significant increases. To counteract α error accumulation, we used the conservative Bonferroni correction,^9^ which is why the significance level was adapted to α* = *α*/*n*, with *n* as the number of tests.

Because the sample size was too small for a statistical evaluation for both vascular and functional diseases, only the statistical calculations for the malignant and benign tumors were analyzed.

## Results

**Fig. 1:**
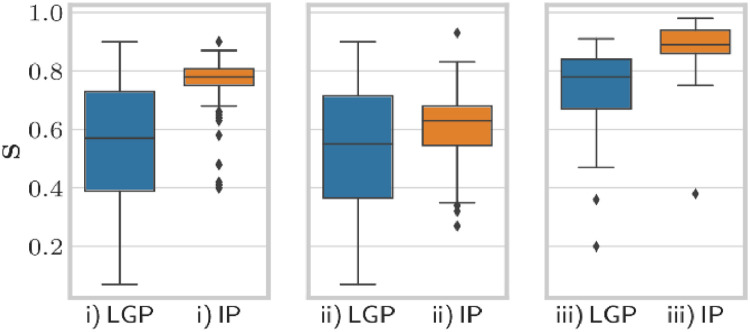
Box plots for parameter selectivity dependent on the inverse planning strategy including all cases. i), “maximize coverage, favor selectivity”, ii), “maximize coverage, favor beam-on time”, iii), “maximize selectivity”.

Table 2 provides the summary statistics of the performed *t-*tests dependent on the IP planning strategy, including *P* values for each parameter and the mean difference (per TV) between the respective IP and LGP plan. Exemplary box plots in Figure 1 illustrate the distributions of the parameter selectivity.

**Table 2:** Results with IP plans for the default single run depending on the strategy

*Abbreviations:* BOT = beam-on time; *C* = coverage; *D*_max_ = maximum dose; *D*_mean_ = mean dose; *D*_min_ = minimum dose; EI = efficiency index; GI = gradient index; Gy = gray;  = “maximize coverage, favor selectivity”; ii = “maximize coverage, favor BOT”; iii = “maximize selectivity”; IP = inverse planning; *n* = number; PI = prescription isodose; OAR = organ at risk; PCI = Paddick conformity index; PI = prescription isodose; *s* = corrected sample standard deviation; *S* = selectivity; *t*_plan_ = planning or computational time; *t*_total_ = total set-up time; *V*_X Gy_ = volume treated with X Gy; *X*  = mean value of the respective parameter for LGP; *Y*  = mean value for IP.

*P < .05.

†*P* < .01.

‡*P* < .001.

The EI includes *η*_50%_and the efficiency index for multiple targets (*Gη*_12__Gy_). Highlighted rows have a *P* value just below .05 but above α*. Values for *D*_OAR transgression_ should be interpreted with caution because OARs did not exist for every case (eg, metastases). For the strategies i, ii, and iii, 28, 7, and 87 OARs are counted, respectively. Note that sample sizes are different for each strategy and every parameter.

**Fig. 2:**
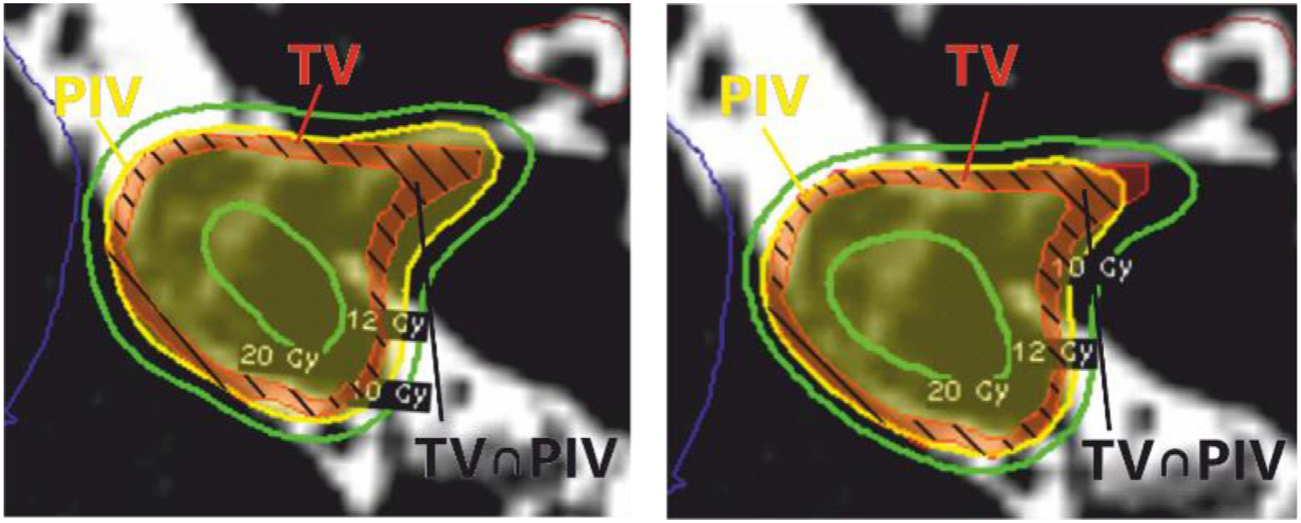
Horizontal T2-weighted magnetic resonance imaging scans demonstrating the comparison between the inverse planning strategy and Leksell GammaPlan for a representative acoustic neuroma case adjacent to the brain stem. Left, Leksell GammaPlan with *C* = 0.99 and *S* = 0.84. Right, inverse planning with *C* = 0.98 and *S* = 0.94. The red-black shaded area represents the intersection of the TV (red) and the 12-Gy prescription isodose (yellow).

On average, we found significantly higher selectivity and PCI values for all available IP planning strategies with high significance (< .001). These parameters improved remarkably for the selectivity strategies with a lower variance, as can be seen from the box plots in Figure 1. This is additionally visualized in Figure 2, in which the inversely optimized isodose is closer to the tumor outline than for the conventional planning method.

The mean IP coverage values were barely distinguishable from the LGP coverage values, only inferior for the strategy prioritizing selectivity. The same applied to the mean GI values and their variance within the interquartile range, except for the strategy “maximize coverage, favor selectivity.” These results were reflected in an overall improved EI, or for the “maximum coverage, favor BOT” strategy in an equal EI. Although there was a significantly lower *t*_plan_ per case for the strategies that prioritized selectivity, IP was slightly inferior to LGP in terms of BOT. These differences almost balanced out again in terms of *t*_total_. Furthermore, the isocenter number increased for all IP strategies. It should be noted that the optimized PI was either significantly below or above the manual adjusted, experience- and knowledge-based PI, independent of the IP strategy. The surrounding tissue, whose protection is characterized by *V*_12 Gy_ or *V*_10 Gy_,^11^^,^^12^ was exposed to approximately the same dose for both planning methods with similar variance, except for the strategy “maximize selectivity.”

In contrast, the inverse planning yielded a high GI for the trigeminal neuralgia compared with LGP. Three more cases with trigeminal neuralgia were not included in the statistical testing, because they were not exportable from IP owing to their high gradient index (>20) or their unsafe declared coverage. For all cases, the calculated PI was much higher than the PI set by the expert planner.

**Fig. 3:**
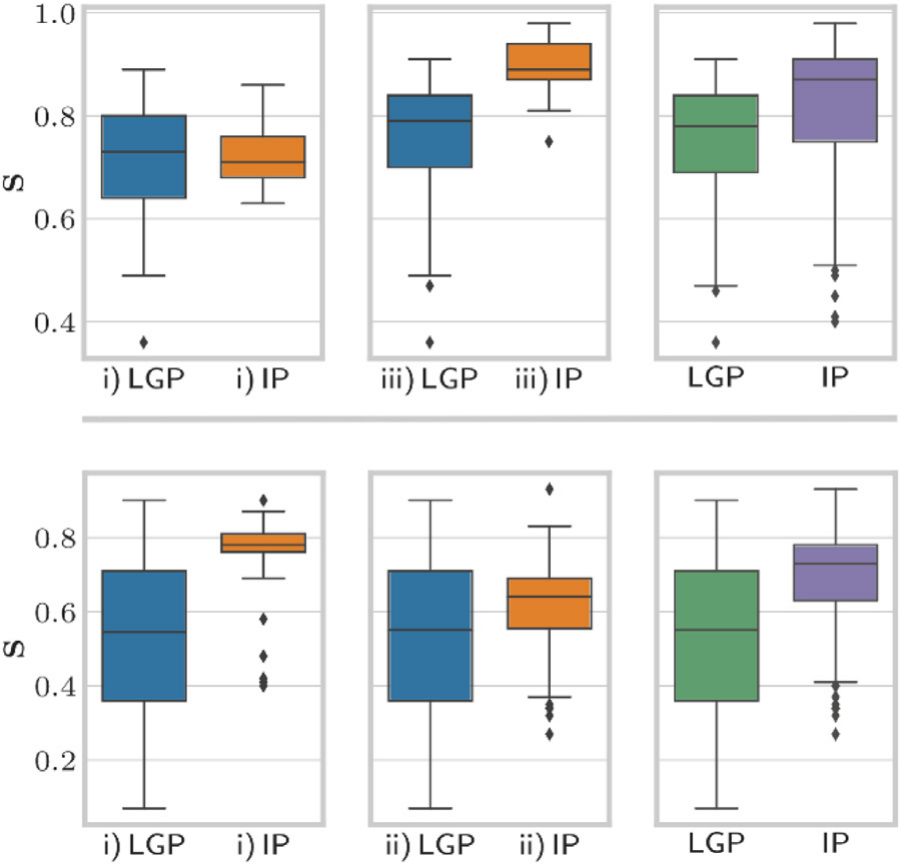
Comparison of benign and malignant entities regarding selectivity. Top, box plots for benign entities depending on the strategy i)/ii)/iii) (same notation as in Fig 1). Bottom, box plots for malignant entities.

**Fig. 4:**
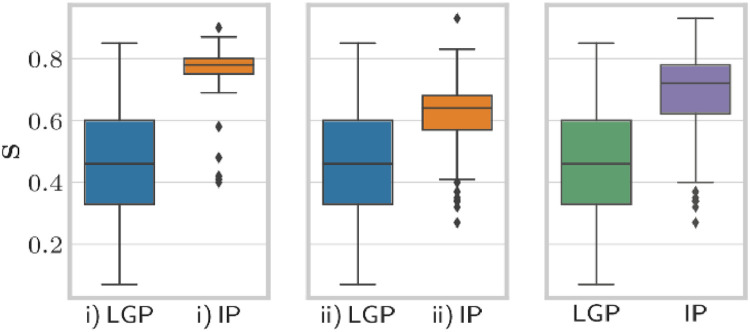
Results for micrometastases regarding selectivity. Box plots for micrometastases (with 1-mm safety margin) depending on the strategy i) and ii) (same notation as in Fig 1).

We additionally tested the performance of the inverse optimization for benign and malignant diseases separately (see Fig 3, Table B1,B2, and Fig A2). Figure 4 specifically shows the selectivity distribution for micrometastases with a volume <1 cm^3^_._

## Discussion

The results of our prospective trial with more than 100 cases demonstrated that the inverse planning software IntuitivePlan calculated comparable high-quality irradiation plans competing with the conventional forward planning. Significantly higher conformity values, shorter planning time, and better OAR sparing characterized the inverse calculated plans. The study demonstrated that the resulting IP plans with their quality metrics were highly dependent on the chosen strategy.

Compared with forward planning, the strategy “maximize selectivity” provided a significant improvement in conformity and a dose reduction in the brain as well as the respective OAR under reduced planning time. However, the dose gradients stayed the same and the BOT changed slightly. Even though the coverage was significantly lower for IP plans, this strategy was superior to the other 2 strategies used, because the surrounding tissue and the OAR were exposed to a lower dose. Hence, we recommend “maximize selectivity” for benign cases, owing to their irregular shape that requires much experience and planning time. The “maximize coverage, favor selectivity” strategy obtained results similar to those of the selectivity-only optimizing strategy. In addition, the same TV coverage as for LGP and a steeper dose fall-off outside the TV were achieved compared with LGP. However, as substantiated by a significantly higher number of shots, the BOT was much longer, which is known to reduce the biologically effective dose^13^ when treating malignant tumors.

To improve the coverage for the final plan, either the IP preset for the minimum selectivity could be changed before computing or the PI could be adapted after the optimization and the back-import into LGP. In most cases, we finally adapted the PI to achieve an even higher plan quality than the first-run IP preplans with the default optimization settings. Alternatively, the dosimetrist could manually adjust the isocenter configuration and the PI in the graphical user interface of IP.

A striking and contrasting result was the large difference in the number of blocked sectors. In general, manual planning includes more blocked sectors to spare radiosensitive tissue. Nevertheless, as the IP results revealed, there was no loss in the OAR protection without any blocked sectors.

The PI differed significantly for all strategies, which consequently influenced the *D*_max_ in the TV. Significantly higher isodoses simultaneously reduced the maximum doses (see the strategy “maximum selectivity”). In Gamma Knife radiation surgery, the PI for manual forward planning is usually the 50% isodose because this is where the steepest dose gradients can be expected.^14^ The effect of a higher or lower *D*_max_ on the treatment outcome could not be confirmed, so that a change of this is quite legitimate.^15^^,^^16^

Upon closer examination of the benign diseases, IP was only better than LGP in GI and barely better in *t*_plan_ and *D*_OAR transgression_. With respect to coverage, conformity, and sparing of surrounding tissue, IP was as good as LGP. The PI, by contrast, was comparatively higher for the same prescription dose so that the *D*_max_ in the target was lower. As a consequence, the reduced *D*_max_ could decline the desired ablative effect of single-time irradiation of benign tumors. The dose-volume data of normal brain, a good predictor of radionecrosis, was approximately the same for both planning methods but with higher brain protection for the “maximum selectivity” strategy. Further reduction would still be desirable. According to Blonigen,^8^ the risk to develop radionecrosis would be already 34% for measured values of 6.4 to 14.5 cm^3^ and 4.8 to 10.8 cm^3^ in the *V*_10 Gy_ and *V*_12 Gy_ distribution, respectively.

The so-called “one-shot plans,” which were created manually for multiple metastases and micrometastases, did show advantages compared with IP plans. Although the target conformity was much higher for IP plans optimized with “maximum coverage, favor selectivity” than that for LGP, the most important goal for these malignant cases is the TV coverage, followed by a short BOT for patients with a poor state of health. Because the strategy “maximum coverage, favor selectivity” primarily focuses on conformity, the BOT is considerably increased. For multiple metastases, the strategy “maximize coverage, favor BOT” seems to be most suitable but often is not preferable to the standard manual method in which a single isocenter with the smallest possible collimator is used. For micrometastases, the surrounding brain tissue receives little dose, even with high coverage. Thus, a lower selectivity of manual single-shot plans would be less clinically relevant. More selective plans, generated by IP as default, would lead to an increase in BOT owing to a higher number of shots.

Previous work by Régis et al^17^ and Paddick et al^18^ focused primarily on ANs and AVMs that require maximum selectivity. In our trial, however, the small sample size of AVMs hampered meaningful conclusions. In contrast to these previous studies,^17^^,^^18^ the IP strategy was not the same for all cases and varied depending on the entity. The significantly higher number of shots among IP plans, which is contrary to usual expert planning, is consistent with Paddick et al and Régis et al.^17^^,^^18^ However, we could not verify the strong influence of η_shots_ on the BOT. The nonsuperiority of the GI (except for the strategy “maximize coverage, favor selectivity”) is also consistent with former studies. Our study does not (entirely) confirm previous results regarding selectivity and PCI, because these studies achieved higher conformity values for their manual plans than did our manual preplans (*S* = 0.910 ± 0.074, PCI = 0.898 ± 0.076 in the study by Régis et al; *S* = 0.856, PCI = 0.824 in the study by Paddick et al). Paddick assumed a positive correlation between BOT and PCI.^18^ We showed that these parameters correlated depending on the planning method (*r*_Pearson_ = 0.098, 0.32, and –0.03 for “maximize coverage, favor selectivity,” “maximize coverage, favor BOT,” and “maximize selectivity,” respectively). The ANs represent one of the most challenging indications in stereotactic radiation surgery, even though the GK was originally developed for such purposes in 1969.^19^^,^^20^ Our study included 21 AN cases for which 20 IP plans were preferred with the strategy “maximize selectivity” owing to comparatively higher selectivity (IP: 0.89 ± 0.06 vs LGP: 0.74 ± 0.14) despite lower coverage (IP: 0.96 ± 0.04 vs LGP: 0.987 ± 0.007). Both previous studies^17^^,^^18^ did not verify the superiority of IP in OAR and brain sparing. Especially for the strategy “maximize selectivity,” our findings contrast with these results. The underlying reason is that in both studies,^17^^,^^18^ no preplans, but highly optimized forward plans, were used for the comparison with IP.

The planner's assessment and experience considerably influence the resulting plans, especially for GK centers with less experienced in stereotaxis personnel. Moreover, further improvements in the clinically acceptable LGP preplans (eg, higher conformity) were certainly possible but had to be weighed against the planning time. As noted, the compared plans were preliminary. Staffed with more dosimetrists and equipped with a stereotactic planning MRI allowing for planning directly on stereotactic MRI images, many GK facilities routinely do not use preplanning. Our methods are therefore more suitable for smaller institutions. If preplanning, the planner can spend more time on a specific case, compare different plan versions, and then select the optimal plan, which removes the time pressure on the treatment day as the patient is waiting with the frame attached. For teams with medium experience in stereotaxis, such an algorithm provides a plan alternative or basis.

Unlike nonstereotactic treatment planning systems, the treatment planning system in Gamma Knife radiation surgery did not include a global algorithm. IntuitivePlan was the first available third-party algorithm for GK. A comparable algorithm, Leksell Gamma Knife Lightning, was offered only later by the GK manufacturer. According to our information, there is no direct relation between the 2 algorithms.^21^ The algorithm used in this study is freely available now, and it has been and is still used in other, nonmedical areas (eg, in the planning of power supply networks) and for scientific purposes. Although the implementation in software compatible with a GK plan is no longer available for purchase, its fundamental aspects of the general use of an optimization algorithm can nevertheless be transferred. First, inverse planning can save much time in planning and make full use of the GK device. Second, if one has the possibility to select different objectives or strategies, several optimized plans can be compared more easily to find the most suitable plan for the respective disease, its stage, and its localization. Further possibilities for manual postplan adaptation or for modification of default optimization settings, as provided by IP, allow for taking the anamnesis into account.

## Conclusions

The inverse planning algorithm achieved clinically acceptable preplans for all planning strategies within a reasonable time and with at least equal or superior quality compared with LGP. Therefore, the algorithm proves beneficial in clinical routine, especially for smaller GK facilities with less experienced planners. Our findings suggest that inverse planning is generally appropriate for complex-shaped tumors and that forward planning, by contrast, is suitable for trigeminal neuralgia or micrometastases.

## Appendix Supplementary materials

Image, application 1

Image, application 2

## Footnotes

Supplementary material associated with this article can be found in the online version at doi:10.1016/j.adro.2022.101006.
