# Supplementary material for: Evaluation of a New Inverse, Globally Convex Treatment Planning System Algorithm for Gamma Knife Radiation Surgery Within a Prospective Trial: Advantages and Disadvantages in Practical Application
Source: Adv Radiat Oncol. 2022 Jun 29;7(6):101006. doi: 10.1016/j.adro.2022.101006 (PMC9436708; doi:10.1016/j.adro.2022.101006)
Supplement: Supplementary file 1 [file mmc1.pdf]

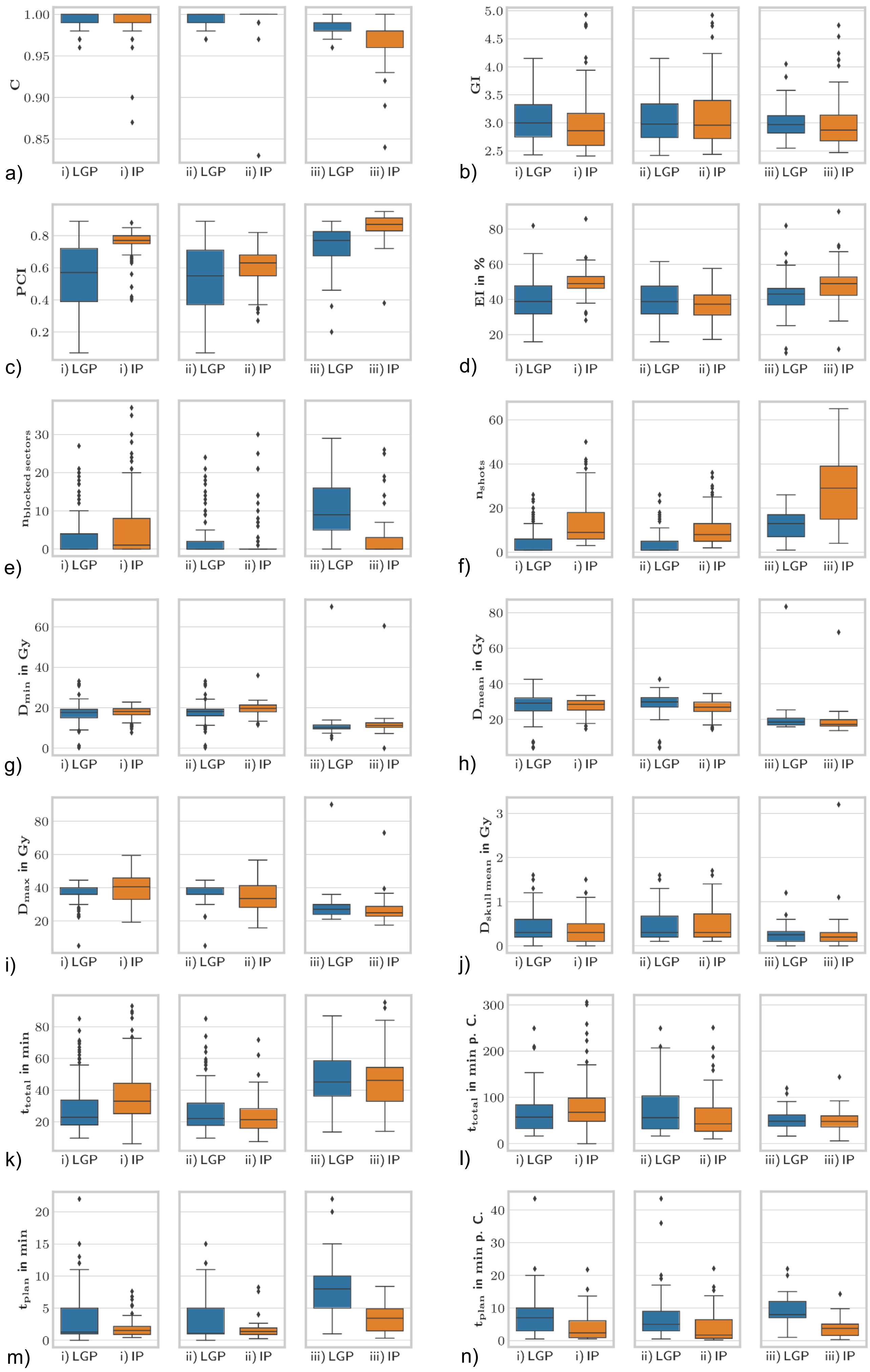
a)
C
b)
GI
c)
PCI
d)
EI in %
e)
$n_{blocked\ sectors}$
f)
$n_{shots}$
g)
$D_{min}$ in Gy
h)
$D_{mean}$ in Gy
i)
$D_{max}$ in Gy
j)
$D_{skull\ mean}$ in Gy
k)
$t_{total}$ in min
l)
$t_{total}$ in min p. C.
m)
$t_{plan}$ in min
n)
$t_{plan}$ in min p. C.
i) LGP
i) IP
ii) LGP
ii) IP
iii) LGP
iii) IP

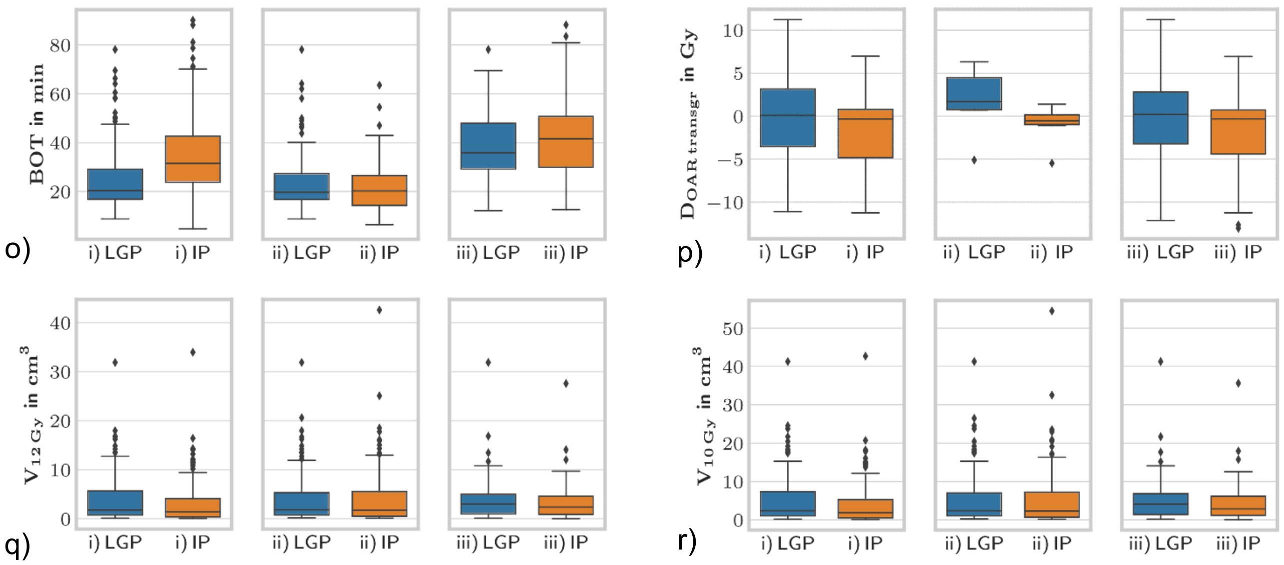


**Fig. A1a)-r)** Box plots for the investigated parameters dependent on the IP strategy i)/ ii)/ iii).

*Abbreviations:* i) "maximize coverage favor selectivity"; ii) "maximize coverage favor BOT"; iii) "maximize selectivity".

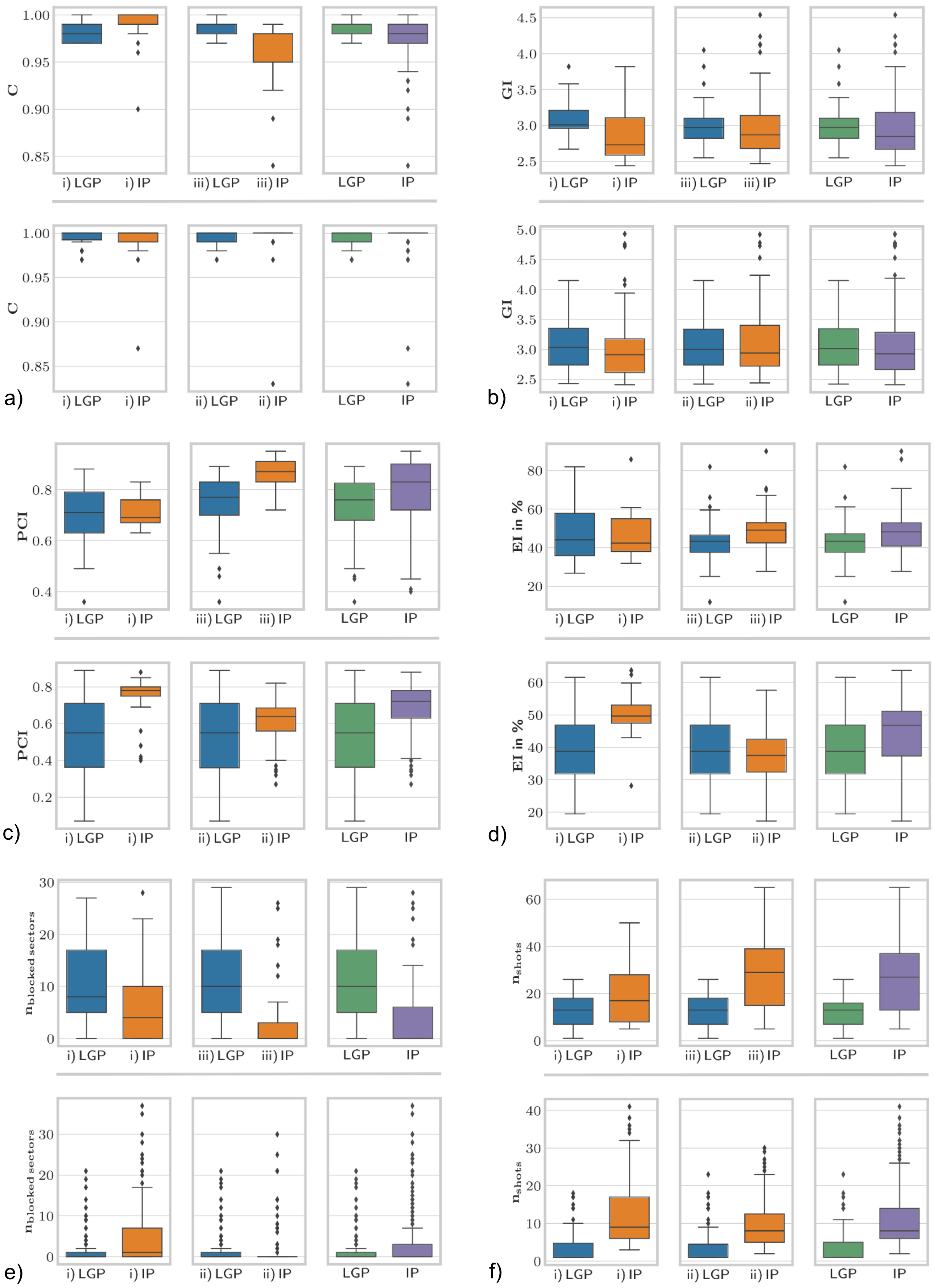

a)
C
1.00
0.95
0.90
0.85
i) LGP
i) IP
iii) LGP
iii) IP
ii) LGP
ii) IP
LGP
IP
b)
GI
c)
PCI
d)
EI in %
e)
$n_{blocked\ sectors}$
f)
$n_{shots}$

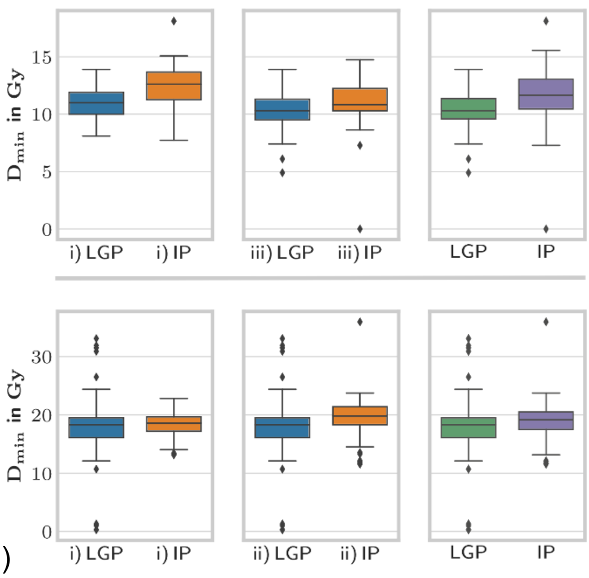
$D_{min}$ in Gy
i) LGP
i) IP
iii) LGP
iii) IP
LGP
IP
$D_{min}$ in Gy
i) LGP
i) IP
ii) LGP
ii) IP
LGP
IP

g)

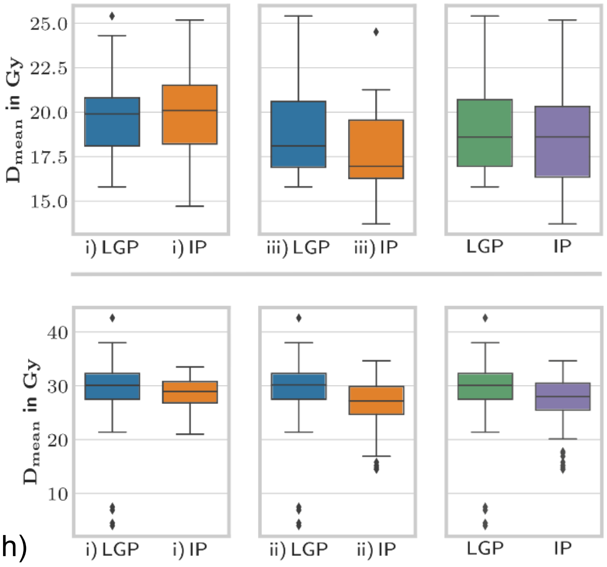
$D_{mean}$ in Gy
i) LGP
i) IP
iii) LGP
iii) IP
LGP
IP
$D_{mean}$ in Gy
i) LGP
i) IP
ii) LGP
ii) IP
LGP
IP

h)

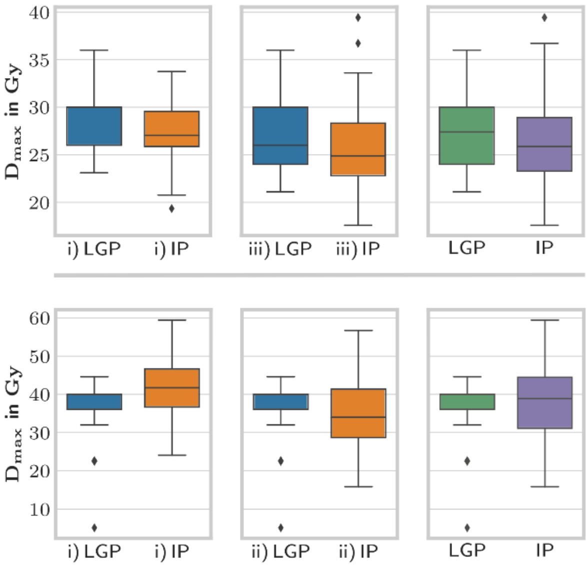
$D_{max}$ in Gy
i) LGP
i) IP
iii) LGP
iii) IP
LGP
IP
$D_{max}$ in Gy
i) LGP
i) IP
ii) LGP
ii) IP
LGP
IP

i)

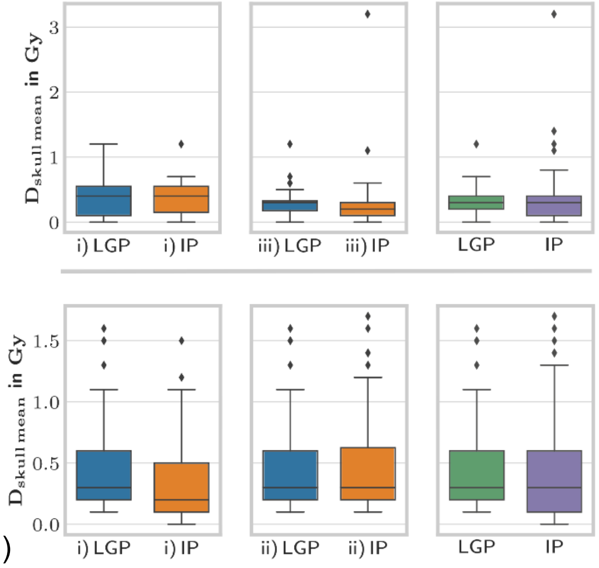
$D_{skull\ mean}$ in Gy
i) LGP
i) IP
iii) LGP
iii) IP
LGP
IP
$D_{skull\ mean}$ in Gy
i) LGP
i) IP
ii) LGP
ii) IP
LGP
IP

j)

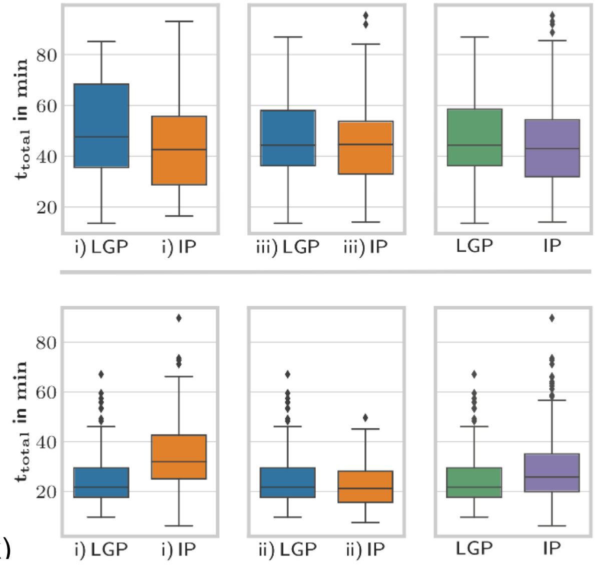
$t_{total}$ in min
i) LGP
i) IP
iii) LGP
iii) IP
LGP
IP
$t_{total}$ in min
i) LGP
i) IP
ii) LGP
ii) IP
LGP
IP

k)

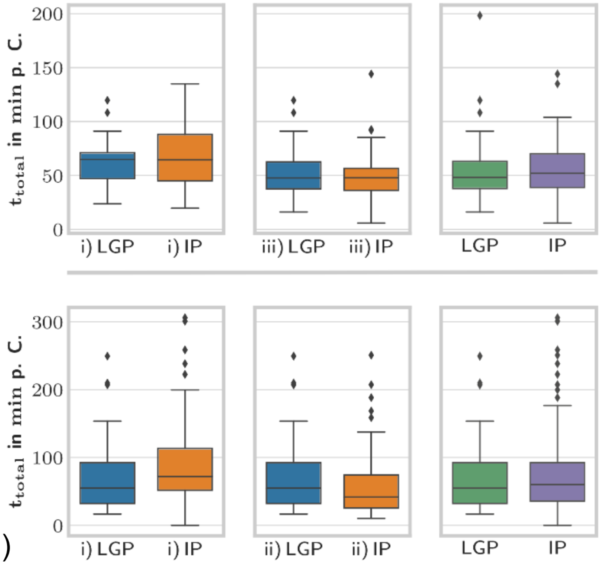
$t_{total}$ in min p. C.
i) LGP
i) IP
iii) LGP
iii) IP
LGP
IP
$t_{total}$ in min p. C.
i) LGP
i) IP
ii) LGP
ii) IP
LGP
IP

l)

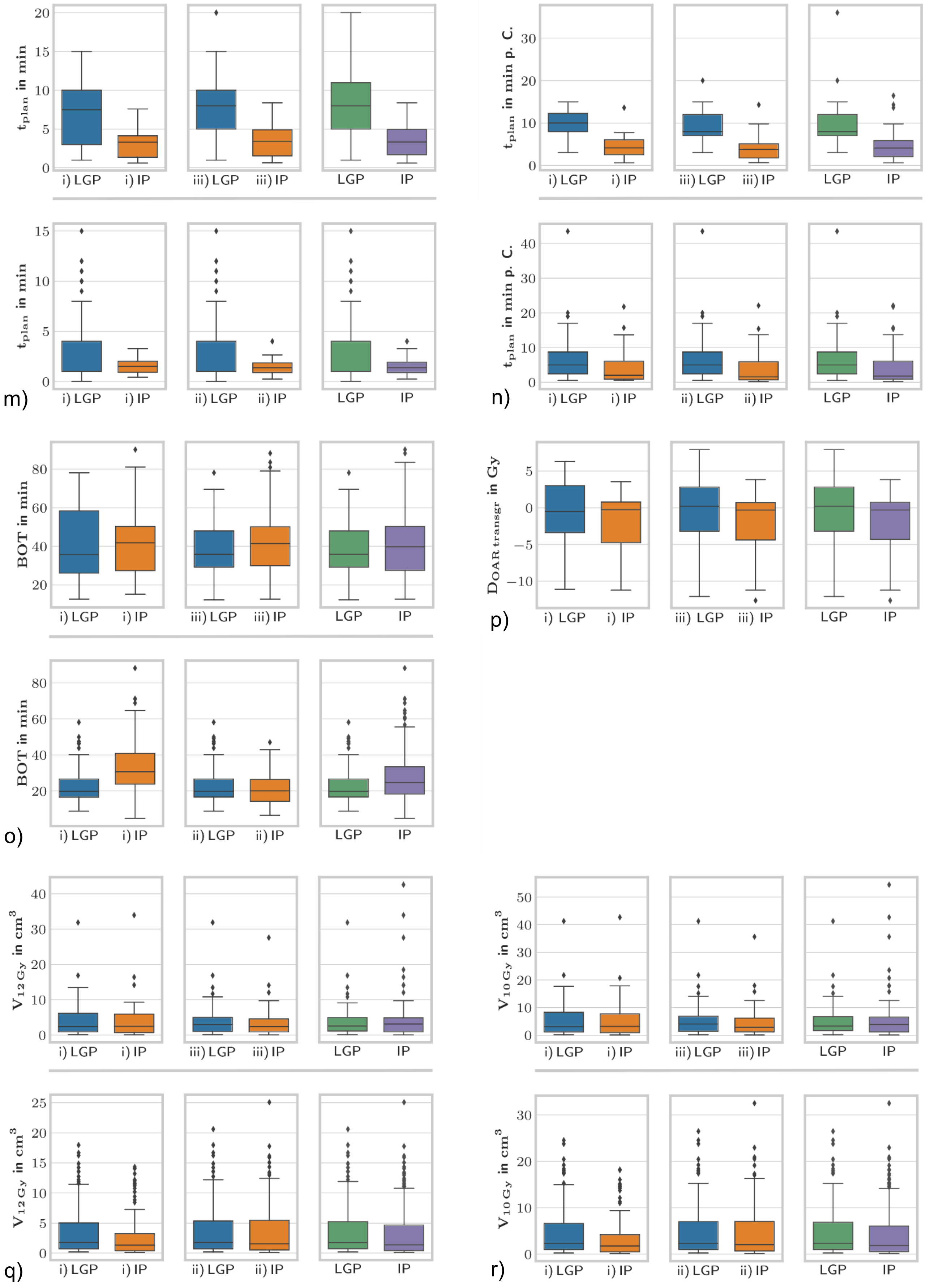


**Fig. A2a)-r)** Comparison between benign and malign entities for the investigated parameters.

The upper box plots of the respective subfigures (a)-r)) represent the results for benign entities (meningiomas, neurinomas, adenomas and ACNs) and the lower boxplots for malignant disorders (singular and multiple cerebral (micro-)metastases). The left and middle box plots for each subfigure display the outcomes according to the different strategies (same notation as in Figure A1). and the right box plots for each subfigure regardless of the strategy. As no OARs were considered in the treatment planning for malign entities, the parameter $D_{OAR\ transgression}$ is not indicated. *Abbreviations:* i) "maximize coverage favor selectivity"; ii) "maximize coverage favor BOT"; iii) "maximize selectivity".

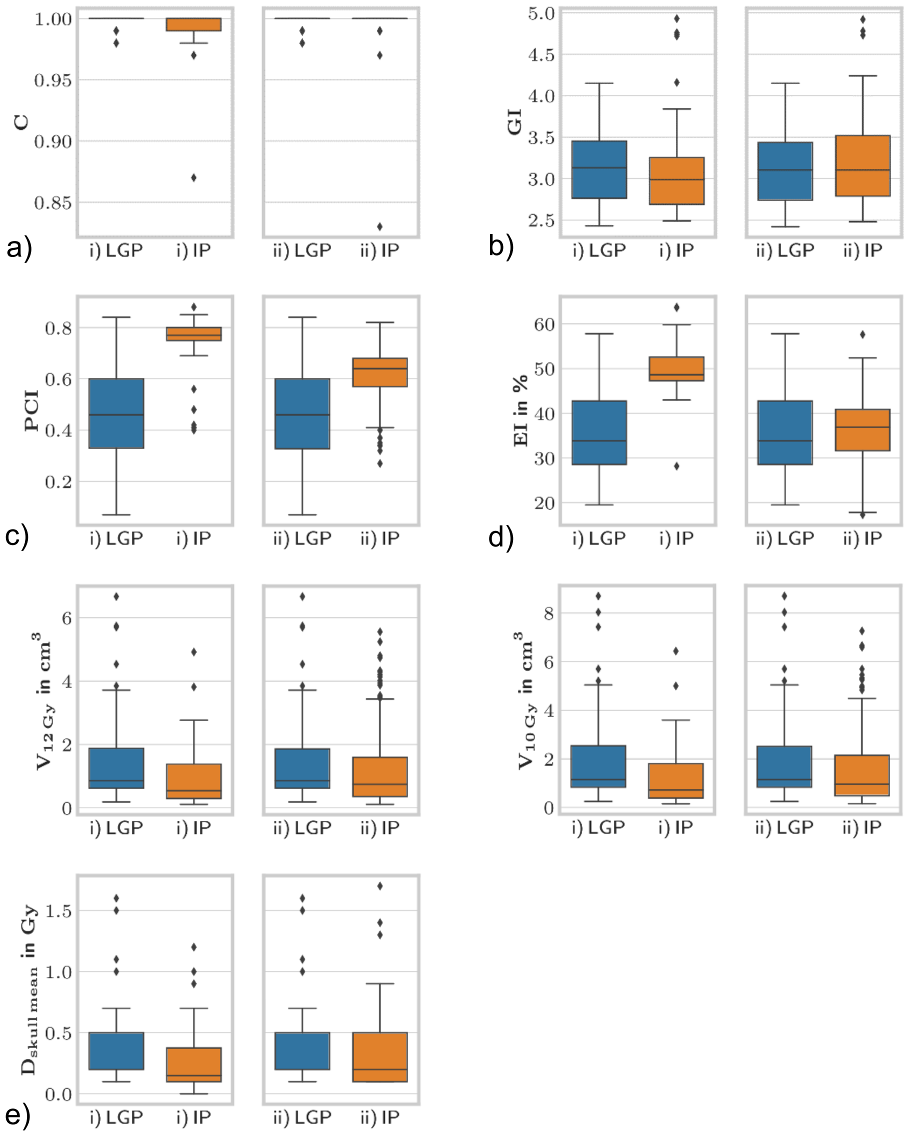


**Fig. A3a)-e)** Results for micrometastases.

*Abbreviations:* i) "maximize coverage favor selectivity"; ii) "maximize coverage favor BOT”
