# Supplementary material for: Evaluation of a New Inverse, Globally Convex Treatment Planning System Algorithm for Gamma Knife Radiation Surgery Within a Prospective Trial: Advantages and Disadvantages in Practical Application
Source: Adv Radiat Oncol. 2022 Jun 29;7(6):101006. doi: 10.1016/j.adro.2022.101006 (PMC9436708; doi:10.1016/j.adro.2022.101006)
Supplement: Supplementary file 2 [file mmc2.pdf]

Table B1 Plan analysis of benign tumors

Parameter	Strategy	LGP $\bar{X} \pm s$	IP $\bar{Y} \pm s$	$(\overline{X - Y}) \pm s$	p ($\alpha = 0.05$)	Significance
PI in %	i)	49.9 ± 0.8	52 ± 5	-2 ± 5	0.0277^{*}	yes
	iii)	50.2 ± 1.4	53 ± 8	-3 ± 8	0.0008^{***}	yes
C	i)	0.983 ± 0.011	0.989 ± 0.024	-0.006 ± 0.029	0.3263	no
	iii)	0.984 ± 0.008	0.966 ± 0.028	0.02 ± 0.03	$1.77 * 10^{-5***}$	yes
S	i)	0.71 ± 0.14	0.72 ± 0.07	-0.02 ± 0.14	0.5798	no
	iii)	0.76 ± 0.12	0.90 ± 0.05	-0.14 ± 0.10	$3.8 * 10^{-16***}$	yes
GI	i)	3.08 ± 0.30	2.9 ± 0.5	0.2 ± 0.4	0.0167^{*}	yes
	iii)	3.01 ± 0.29	3.0 ± 0.5	0.0 ± 0.5	0.7790	no
PCI	i)	0.69 ± 0.13	0.71 ± 0.07	-0.02 ± 0.12	0.4527	no
	iii)	0.74 ± 0.12	0.87 ± 0.06	-0.12 ± 0.09	$5.82 * 10^{-16***}$	yes
EI in %[†]	i)	47 ± 16	47 ± 15	-1 ± 4	0.1975	no
	iii)	43 ± 12	49 ± 12	-6 ± 4	$8.19 * 10^{-15***}$	yes
t_{plan} per case in min	i)	10 ± 4	5 ± 4	5 ± 5	0.0036^{**}	yes
	iii)	9 ± 4	4.1 ± 2.8	5 ± 4	$1.54 * 10^{-10***}$	yes
BOT in min	i)	42 ± 20	44 ± 23	-2 ± 13	0.5637	no
	iii)	39 ± 15	42 ± 18	-3 ± 12	0.0963	no
t_{total} per case in min	i)	65 ± 29	70 ± 40	-1 ± 15	0.7292	no
	iii)	52 ± 22	51 ± 24	2 ± 15	0.4442	no
n_{shots}	i)	12 ± 8	21.14 ± 10	-9 ± 10	0.0007^{***}	yes
	iii)	13 ± 7	29 ± 15	-16 ± 11	$9.23 * 10^{-17***}$	yes
$n_{blocked\ sectors}$	i)	11 ± 8	6 ± 9	4 ± 13	0.1107	no
	iii)	11 ± 8	4 ± 8	7 ± 11	$3.23 * 10^{-6***}$	yes
$V_{12\ Gy}$ in cm^3	i)	5 ± 8	6 ± 8	-0.1 ± 0.7	0.4409	no
	iii)	4 ± 6	4 ± 5	0.6 ± 0.8	$5.39 * 10^{-7***}$	yes
$V_{10\ Gy}$ in cm^3	i)	7 ± 10	7 ± 10	0.1 ± 0.7	0.4354	no
	iii)	6 ± 7	5 ± 6	0.9 ± 1.2	$3.64 * 10^{-7***}$	yes
$D_{skull\ mean}$ in Gy	i)	0.4 ± 0.4	0.4 ± 0.4	-0.01 ± 0.04	0.1643	no
	iii)	0.28 ± 0.22	0.3 ± 0.5	-0.0 ± 0.5	0.5521	no
D_{min} in Gy	i)	11.0 ± 1.7	12.5 ± 2.2	-1.6 ± 1.8	0.0006^{***}	yes
	iii)	10.3 ± 1.7	11.0 ± 2.1	-0.6 ± 2.1	0.0234^{*}	yes
D_{mean} in Gy	i)	19.8 ± 2.3	19.8 ± 2.7	-0.1 ± 1.6	0.7772	no
	iii)	18.7 ± 2.2	17.7 ± 2.3	0.9 ± 1.4	$5.62 * 10^{-6***}$	yes
D_{max} in Gy	i)	28 ± 4	27 ± 4	1.0 ± 2.3	0.0470^{*}	yes
	iii)	27 ± 4	26 ± 5	1 ± 4	0.0376^{*}	yes
$D_{OAR\ transgression}$ in Gy	i)	-1 ± 6	-2 ± 5	1.2 ± 2.0	0.0054^{**}	yes
	iii)	0 ± 5	-2 ± 4	1.5 ± 1.7	$2.14 * 10^{-12***}$	yes

Abbreviations: i) = "maximize coverage, favor selectivity"; iii) "maximize selectivity"; $\bar{X}$ = mean value for LGP; $\bar{Y}$ = mean value for IP; s = corrected sample standard deviation.

[†] EI includes $\eta_{50\,\%}$ and $G\eta_{12\ Gy}$.

$^{*}p < 0.05$; $^{**}p < 0.01$; $^{***}p < 0.001$.

Marked in red are those rows that have a p-value just below $\alpha = 0.05$ but above α^{*}.

Table B2 Plan analysis of malign tumors

Parameter	Strategy	LGP $\bar{X} \pm s$	IP $\bar{Y} \pm s$	$(\overline{X - Y}) \pm s$	p ($\alpha = 0.05$)	Significance
PI in %	i)	52 ± 7	48 ± 9	4 ± 12	$5.29 * 10^{-5***}$	yes
	ii)	51 ± 7	56 ± 12	-5 ± 13	$7.56 * 10^{-6***}$	yes
C	i)	0.997 ± 0.007	0.995 ± 0.013	0.001 ± 0.015	0.2496	no
	ii)	0.997 ± 0.007	0.998 ± 0.014	-0.00 ± 0.02	0.1800	no
S	i)	0.53 ± 0.21	0.77 ± 0.08	-0.24 ± 0.21	$2.04 * 10^{-31***}$	yes
	ii)	0.53 ± 0.22	0.61 ± 0.12	-0.08 ± 0.25	$3.76 * 10^{-5***}$	yes
GI	i)	3.1 ± 0.5	3.0 ± 0.5	0.2 ± 0.6	0.0012^{**}	yes
	ii)	3.1 ± 0.5	3.1 ± 0.5	0.0 ± 0.6	0.5377	no
PCI	i)	0.53 ± 0.21	0.77 ± 0.08	-0.24 ± 0.21	$6.32 * 10^{-32***}$	yes
	ii)	0.53 ± 0.21	0.61 ± 0.11	-0.08 ± 0.24	$2.01 * 10^{-5***}$	yes
EI in %†	i)	39 ± 11	51 ± 6	-13 ± 10	$3.33 * 10^{-10***}$	yes
	ii)	39 ± 11	37 ± 11	1 ± 11	0.4734	no
t_{plan} per case in min	i)	7 ± 7	4 ± 5	1 ± 6	0.1561	no
	ii)	7 ± 7	4 ± 5	1 ± 6	0.0919	no
BOT in min	i)	22 ± 9	33 ± 14	-11 ± 15	$2.18 * 10^{-18***}$	yes
	ii)	22 ± 10	21 ± 9	1 ± 12	0.1355	no
t_{total} per case in min	i)	70 ± 60	90 ± 70	-32 ± 50	$6.06 * 10^{-5***}$	yes
	ii)	70 ± 60	61 ± 60	2 ± 40	0.7225	no
n_{shots}	i)	3 ± 4	12 ± 9	-9 ± 8	$1.56 * 10^{-30***}$	yes
	ii)	3 ± 5	10 ± 7	-7 ± 7	$2.39 * 10^{-29***}$	yes
$n_{blocked\ sectors}$	i)	2 ± 4	5 ± 8	-3 ± 9	$1.5 * 10^{-6***}$	yes
	ii)	2 ± 4	2 ± 5	-0 ± 7	0.7665	no
$V_{12\ Gy}$ in cm^3	i)	4 ± 10	3 ± 4	1 ± 9	0.0749	no
	ii)	4 ± 10	4 ± 5	0 ± 9	0.5470	no
$V_{10\ Gy}$ in cm^3	i)	5 ± 6	3 ± 5	0.9 ± 2.9	0.0003^{***}	yes
	ii)	5 ± 6	5 ± 6	-0 ± 4	0.3827	no
$D_{skull\ mean}$ in Gy	i)	0.4 ± 0.4	0.4 ± 0.4	0.04 ± 0.17	0.1322	no
	ii)	0.5 ± 0.4	0.5 ± 0.5	-0.05 ± 0.16	0.0577	no
D_{min} in Gy	i)	18 ± 5	18.4 ± 1.8	-0 ± 5	0.2483	no
	ii)	18 ± 5	19.5 ± 3.0	-2 ± 5	$8.58 * 10^{-5***}$	yes
D_{mean} in Gy	i)	29 ± 6	28.5 ± 3.0	1 ± 6	0.0975	no
	ii)	29 ± 6	27 ± 5	2 ± 6	$1.96 * 10^{-6***}$	yes
D_{max} in Gy	i)	38 ± 5	42 ± 8	-4 ± 9	$7.67 * 10^{-8***}$	yes
	ii)	38 ± 5	35 ± 9	3 ± 10	0.0001^{***}	yes

Abbreviations: i) = "maximize coverage, favor selectivity"; ii) "maximize coverage, favor BOT"; $\bar{X}$ = mean value for LGP; $\bar{Y}$ = mean value for IP; s = corrected sample standard deviation.

† EI includes $\eta_{50\ \%}$ and $G\eta_{12\ Gy}$.

$^{*} p < 0.05$; $^{**} p < 0.01$; $^{***} p < 0.001$.
Marked in red are those rows that have a p-value just below $\alpha = 0.05$ but above α^{*}.
